# Intradural Lumbar Disc Herniation From the Lateral Inner Surface of the Dura Without a Penetration Hole: A Case Report

**DOI:** 10.7759/cureus.22418

**Published:** 2022-02-20

**Authors:** Naoki Segi, Kei Ando, Hiroaki Nakashima, Masaaki Machino, Shiro Imagama

**Affiliations:** 1 Orthopaedics, Nagoya University Graduate School of Medicine, Nagoya, JPN

**Keywords:** posterior longitudinal ligament, paraplegia, cauda equina syndrome, lumbar disc herniation, intradural disc herniation

## Abstract

Intradural disc herniation (IDH) is usually diagnosed during surgery when a herniated mass is found to have penetrated the ventral dura. We experienced a case of IDH that entered the dura from the lateral side with no penetrating hole. A 61-year-old man presented to our institution with left leg pain of two months' duration. Plain x-ray imaging showed degeneration of the lumbar spine, and a magnetic resonance imaging (MRI) scan revealed a suspected tumor at the L3-L4 level. Two weeks later, the patient suffered from acute cauda equina syndrome. A gadolinium-enhanced MRI showed an enlarged lesion with no enhancement visible, and emergency surgery was performed. The lesion originated from the left side of the dura. Despite the white debris suggesting a herniated disc, no penetrating hole was found in the dura. Pathologically, the lesion was found to be an intervertebral disc and was diagnosed as an intradural lumbar disc herniation. The patient’s neurological symptoms improved, but he did not recover his left ankle dorsiflexion. In a degenerated lumbar spine, IDH may not always originate from the ventral dura and may not be accompanied by a penetrating hole.

## Introduction

Intradural disc herniation (IDH) is a rare condition that is difficult to diagnose on preoperative magnetic resonance imaging (MRI). It is usually confirmed at the time of surgery when a herniated mass is found to have entered the ventral dura. Herein, we report a case of an intradural prolapse of a herniated disc from the lateral side, rather than the ventral dura, with no apparent penetration hole.

## Case presentation

**Figure 1:**
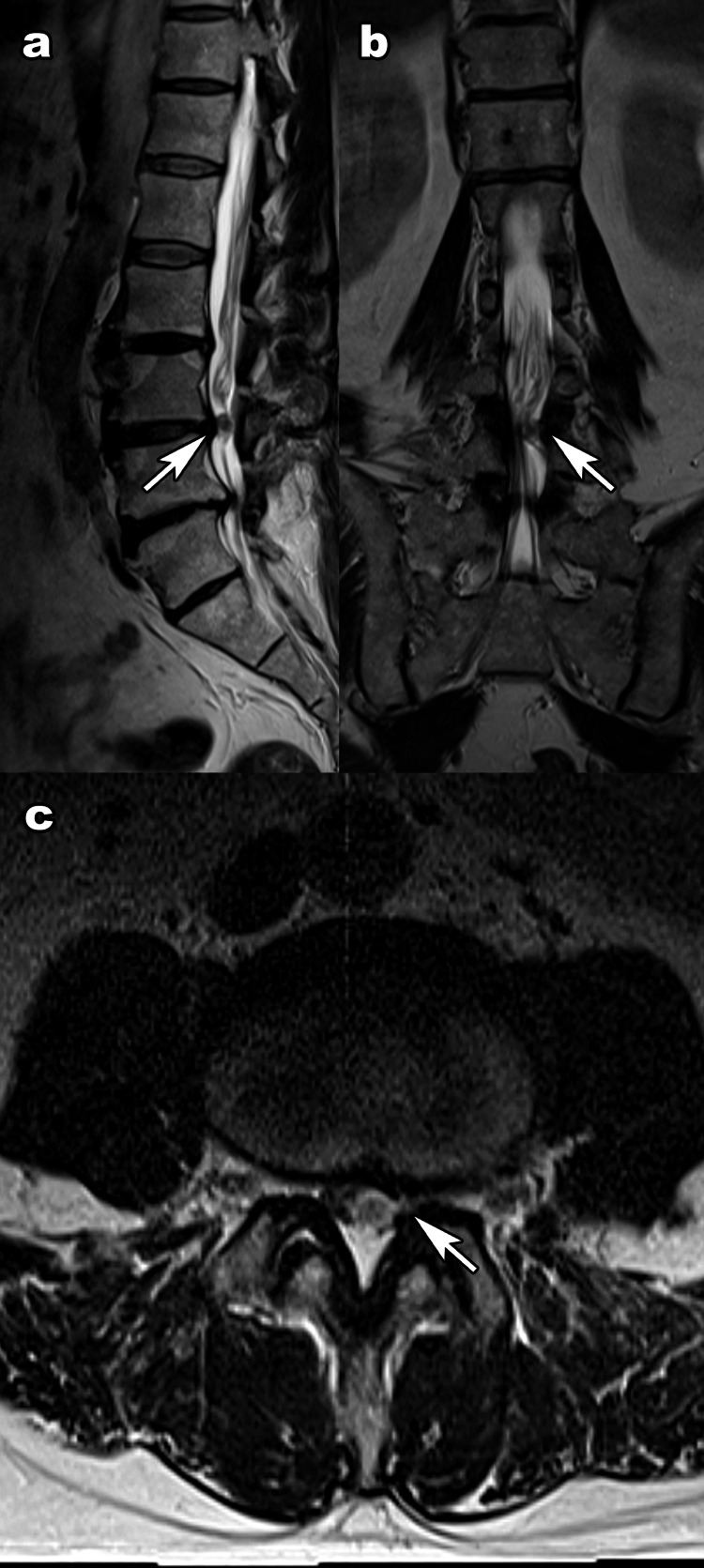
Initial magnetic resonance imaging. T2-weighted images suggested an intradural lesion at the L3-L4 level. (a) Sagittal images showed no obvious discontinuity of the posterior longitudinal ligament (arrow). (b) Coronal images showed an unclear relationship between the lesion and the dura (arrow). (c) In retrospect, this transverse image suggested lateral dural discontinuity (arrow).

A 61-year-old man presented to our institution complaining of intermittent left leg pain that had begun two months earlier. The patient had a history of lung cancer and type 2 diabetes mellitus. Walking caused pain from the left buttock to the outside of the left thigh, which dissipated when he rested. Muscle strength and deep tendon reflex tests were normal, and there was no bladder or bowel dysfunction. Plain x-ray images showed degeneration of the lumbar spine, and an MRI found stenosis at the L3-L4 level due to a suspected tumor (Figure 1).

**Figure 2:**
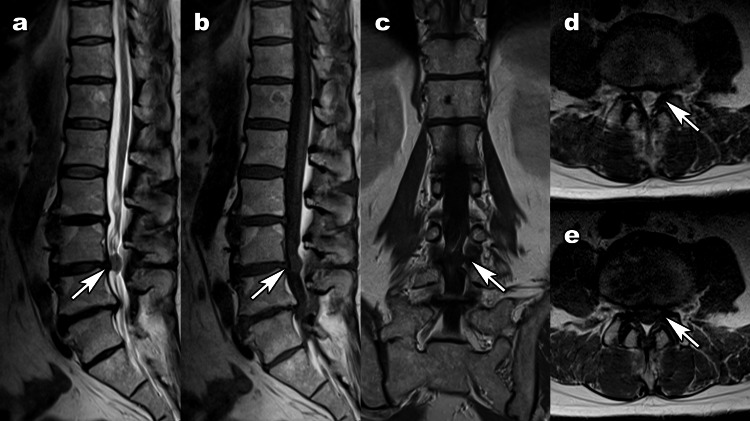
Magnetic resonance imaging after the onset of cauda equina syndrome. (a) T2-weighted sagittal images showed enlargement of the lesion but no discontinuity of the posterior longitudinal ligament (arrow). (b), (c), and (e) No gadolinium enhancement was observed. (d) T2-weighted transverse images showed worsening cauda equina compression.

A contrast-enhanced MRI scan was therefore planned for further evaluation. However, two weeks later, the pain in the patient’s left leg became severe with worsening low back pain, and at the same time, the left ankle joint became impossible to dorsiflex, despite none of the usual triggers. This was followed by difficulty walking due to an inability to move both ankles a few hours later. He also had urinary retention. An urgent MRI scan with no gadolinium enhancement showed an enlarged lesion (Figure 2). No abnormalities were apparent on a computed tomography scan.

**Figure 3:**
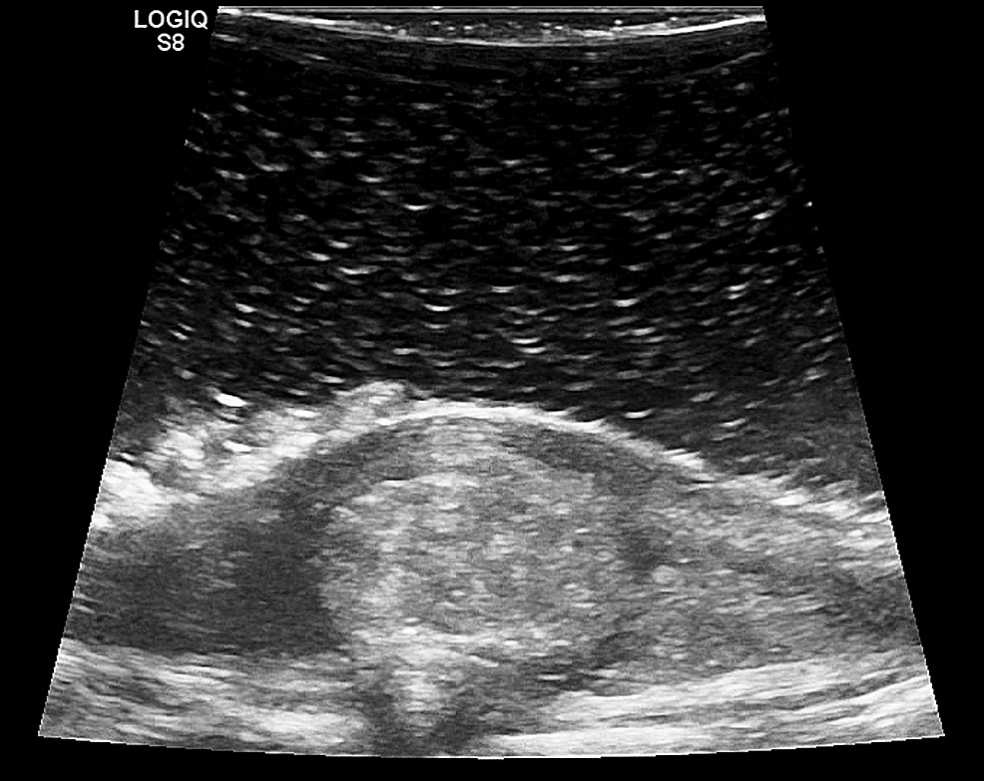
Intraoperative ultrasound findings. In the sagittal plane, the lesion was located at the L3-L4 level, but there was no continuity with the disc. Dura continuity was identified between the lesion and the ventral low echoic region.

An acute cauda equina syndrome was diagnosed, requiring emergency surgery. An L3-L4 laminectomy was performed, followed by ultrasonography to confirm the lesion, which was located at the L3-L4 disc level but was not continuous with the disc (Figure 3).

**Figure 4:**
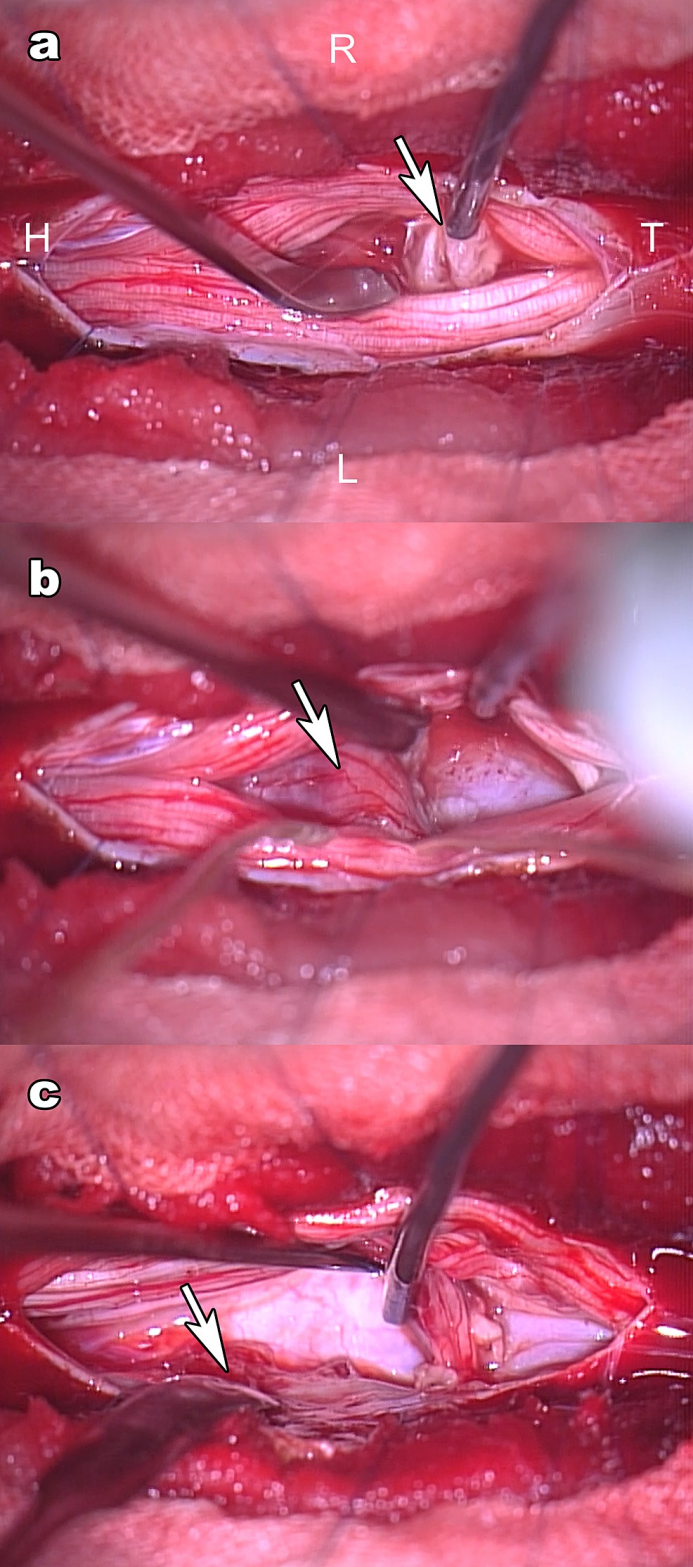
Intraoperative microscopic view. (a) Upon dural incision, white, spongy fragments (arrow) were immediately observed in the subarachnoid space. (b) The left L4 nerve root (arrow) was flattened because of extreme compression from the ventral side. (c) The lesion was found to originate from the inner left wall of the dura (arrow), but no penetrating hole was apparent.

When the dura was incised, white debris was observed. This led us to assume a herniated disc (Figure 4a). The lesion was causing considerable compression of the posterior root of the L4 from the ventral side (Figure 4b). The lesion originated from the left medial surface of the dura (Figure 4c). The lesion's membrane had failed, and the contents were leaking out; therefore, the lesion was reduced in size by enucleation and was removed. No hole was found in the dura (Figure 4c).

**Figure 5:**
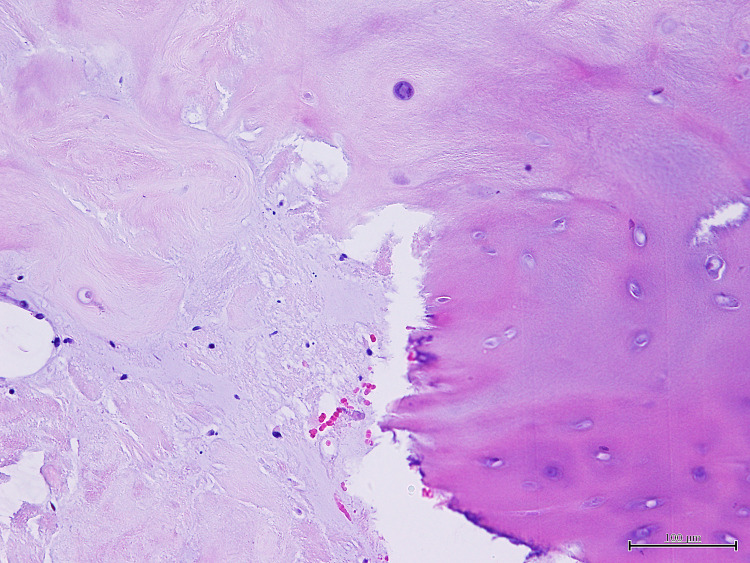
Histological findings. Hematoxylin and eosin staining revealed typical cartilage (objective 20×).

Postoperatively, the patient gradually recovered his muscle strength but did not recover his left ankle dorsiflexion. His bladder regained its function. Histologically, there were no findings of neoplasm, and fibrocartilaginous tissue that could be considered as intervertebral disc was identified (Figure 5). The diagnosis was therefore IDH.

## Discussion

IDH is a rare condition that accounts for 0.26%-0.30% of lumbar disc surgeries [1,2]. There is a higher incidence of cauda equina syndrome in IDH than in epidural disc herniation [3,4]. The etiology of IDH is unknown, but previous studies have postulated that adhesions of the ventral dura and posterior longitudinal ligament (PLL) promote disc herniation into the dural sac [3,5,6]. However, in this case, the IDH did not originate from the ventral dura but from the lateral side. Intraoperatively, IDH was suspected, but no dural defect or hole was found. Furthermore, the lesion had firmly adhered to the lateral inner surface of the dura, leading us to reject our initial suspicion of IDH and form an intraoperative diagnosis of meningioma. The presence of spinal canal stenosis at the L4-L5 level and degeneration at the L3-L4 facet joints suggests that the patient may originally have had spinal canal stenosis at the L3-L4 level as well. Therefore, rather than the adhesion between the PLL and the ventral dura, there may have been an adhesion between the yellow ligament and the lateral dura, and this may have been the location of the herniated disc penetration. However, as the penetration hole was not identified intraoperatively, the IDH may have resulted from an alternative pathogenic factor.

Preoperative diagnosis of IDH is often difficult [5,7,8]. IDH typically has a low signal on both T1- and T2-weighted MRI [3]. Known features of IDH include loss of continuity of the PLL on sagittal images and the “hawk-beak” sign (disc fragments with sharp cartilage endplates) on T2-weighted transverse images [9]. In addition, gadolinium-enhanced MRI may show ring enhancement of the herniated disc area [3,7]. A prolapsed herniated fragment may also be seen in the dura (“crumble disc sign” [10]). However, in the current case, none of these signs were present. At the patient’s initial presentation, the lesion had clear borders, and a low signal on the T1-weighted image and a relatively high signal on the T2-weighted image. Therefore, the differential diagnoses were an intradural extramedullary tumor (schwannoma or meningioma) or a metastatic tumor because the patient had a history of cancer. Additional imaging examinations were scheduled but, unfortunately, the patient’s neurological symptoms worsened rapidly two weeks later. We performed an enhanced MRI scan to investigate possible IDH, hematoma, or intratumoral schwannoma hemorrhage [11]. Although the lesion was found to be enlarged, no new information was obtained because there was no enhancement effect at all, including the absence of ring enhancement. Intraoperative findings ruled out hematoma or schwannoma. Although the lesion contents were suggestive of a herniated disc, we considered it to be a tumor because the capsule of the lesion adhered to the lateral dural wall and there were no holes through which a herniated disc might have penetrated. A definitive diagnosis was finally achieved pathologically.

## Conclusions

We have reported a case of IDH with cauda equina syndrome that could not be definitively diagnosed intraoperatively because of its atypical nature. At the level of the degenerated lumbar spine, IDH may not always originate from the ventral dura and may not be accompanied by an obvious penetration hole. Yet, regardless of how much is known about the lesion, prompt surgery is always necessary for acute-onset cauda equina syndrome.
